# When Being in the Minority Feels Threatening: Social Identity and the Reinforcement of Anti‐Vaccination Attitudes

**DOI:** 10.1111/sjop.70072

**Published:** 2026-01-16

**Authors:** Emma A. Renström, Hanna Bäck, Amanda Remsö

**Affiliations:** ^1^ Kristianstad University Kristianstad Sweden; ^2^ Lund University Lund Sweden

**Keywords:** anti‐vaccination attitudes, intergroup threat, negative emotions, social identity

## Abstract

The present research aims to contribute to the understanding of anti‐vaccination attitudes. We do this by analyzing the role of social identity and intergroup threat. Drawing on intergroup threat theory, we hypothesize that being informed that the general population is positive toward vaccines may be perceived as threatening to individuals identifying as vaccine‐hesitant, which may lead to stronger anti‐vaccination attitudes. We evaluate this hypothesis in two survey experiments performed among Swedish citizens (Study 1, *N* = 376; Study 2, *N* = 698), where we present participants in the treatment group with information that the position toward vaccines in the general population is positive. We find that when vaccine‐hesitant individuals are informed that the general population is positive toward vaccines, they express stronger anti‐vaccination attitudes. We further find that this relationship is mediated by negative emotions, implying perceptions of intergroup threat. We conclude that individuals with a vaccine‐hesitant identity feel threatened when learning that most others are positive toward vaccines, which is associated with stronger anti‐vaccination attitudes. These results have important implications for the understanding of anti‐vaccination attitudes, suggesting that such attitudes may increase when individuals feel that their identity is threatened.

## Key Points

- Vaccine hesitancy functions as a social identity.
- Pro‐vaccination majority norms can feel threatening to vaccine‐hesitant individuals.
- Such threats lead to negative emotional experiences which in turn result in stronger anti‐vaccination attitudes.

## 1 Introduction

In 2019, the World Health Organization (WHO) declared vaccine hesitancy to be one of 10 major threats to global health (2019). Consequently, much research has been dedicated to better understanding the psychological and contextual causes and correlates of vaccine hesitancy. Vaccine hesitancy refers to a delay in the acceptance of vaccination or a refusal of vaccination, including anti‐vaccination attitudes. This article contributes to that research agenda, specifically focusing on social identity and intergroup threat as drivers of anti‐vaccination attitudes. Social identity entails psychological and emotional attachments that individuals feel to different social groups that they belong to (Hornsey 2021; Tajfel and Turner 2004). Research shows that attitudes are sufficient to constitute group boundaries, leading to liking of individuals sharing one's own attitude and dislike of those with other positions (Bäck and Lindholm 2014). Thus, having certain attitudes regarding vaccination, such as anti‐vaccination attitudes, is likely to be associated with sharing a social identity with others with similar attitudes (Motta et al. 2023).

To specify our theoretical argument, we draw on intergroup threat theory, which states that when individuals perceive that other groups can threaten their own social group, for instance, threat to their security or values and beliefs, they become motivated to distance themselves from these other groups and defend their position (Stephan et al. 2015; Renström et al. 2021, 2023). Specifically, we suggest that information that the general population is positive toward vaccination will be perceived as threatening for those highly identified as vaccine‐hesitant, as it contradicts their own attitudes, and that this will lead to higher anti‐vaccination attitudes compared to those lower in such an identity. Such threat perceptions among vaccine‐hesitant individuals may be rooted in “healthism,” or the idea that an individual's health outcomes are solely determined by individual choices, and this is often correlated with anti‐vaccination attitudes (Milionis et al. 2023). Such ideas further tend to co‐occur with perceptions of low risk of disease but high risk of vaccination complications and distrust in health institutions (Kirbiš 2023).

We evaluate our hypotheses in the context of Sweden. Sweden is a country that is characterized by a high confidence in vaccines. When hesitancy is expressed, it mainly concerns new vaccines (Byström et al. 2020). For instance, when Covid‐19 hit, as much as 40% of the Swedish population expressed doubts against vaccination during the first year of the pandemic (Lindvall and Rönnerstrand 2022).

The present study reports the results from two online experiments, where we present participants with information that the general population's position on vaccination is positive. We find that participants who identify as vaccine‐hesitant react with negative emotions to such information, and this reaction is associated with stronger anti‐vaccination attitudes. These results have important implications for understanding anti‐vaccination attitudes since they show that if such attitudes are rooted in an identity position, they may become stronger when individuals feel like their identity is threatened.

## 2 Theoretical Framework

### 2.1 Social Identity Theory and the Role of Perceived Intergroup Threat

Our theoretical argument builds on social identity theory (Tajfel and Turner 2004). Social identity theory argues that an individual's social group(s) become a significant part of their self‐definition through the formation of emotional and psychological bonds to group(s) that one belongs to. Because group belongingness is emotionally important, individuals strive to become or stay members of social groups. The categorization of individuals into socially constructed categories based on attitudes facilitates general inferences about the members of these categories and creates intergroup biases such as liking of ingroup members and dislike of outgroup members (Fiske 1998). Importantly, research finds that simply sharing an attitude with others is sufficient to evoke such intergroup biases (Kenworthy and Miller 2002; Bäck and Lindholm 2014). Thus, attitude positions function to create meaningful social categories and meaningful social group memberships. Hence, vaccine‐hesitant individuals may become emotionally and psychologically attached to the group of “vaccine‐hesitant individuals.” Identifying with vaccine‐hesitant people as a social group constitutes a psychological attachment to other vaccine‐hesitant individuals (Motta et al. 2023). Such social identification is psychologically advantageous because group members derive self‐esteem and a positive self‐view from the group, beyond any ideological motivations or material benefits that may accrue (Hogg and Abrams 1988).

Social groups are central to an individual's self‐definition, thereby influencing cognitions and behavior (Tajfel and Turner 2004). Given that such identities and group membership(s) are important, threats against the group may evoke resistance and intergroup distancing (Stephan et al. 2015). As identification with a social group grows in relevance to the individual, they become increasingly motivated to defend their social identity and invest in the values and future of the group (Tajfel and Turner 2004). As a group becomes an extension of one's identity, the desire to protect the group against external threats increases. Hence, we suggest that a factor that has not received sufficient attention in understanding anti‐vaccination attitudes, as based on social identity theory, is perceived intergroup threat (Stephan et al. 2015).

Based on social identity theory, intergroup threat theory (Stephan et al. 2015) suggests that threats posed by other groups strengthen intergroup emotions and lead to increased intergroup distancing, such as a cementation of one's opinion. An important aspect is that intergroup threats are perceptions of what power the outgroup can have over the ingroup. Hence, when the ingroup perceives that the outgroup may exert power over them, this may lead to intergroup distancing. Such intergroup distancing functions to solidify group boundaries, making clear who belongs to what group and increasing the psychological distance between the groups. One way to achieve this is by strengthening one's own attitude position (Renström et al. 2023). This process thus leads to a polarization of attitudes and obstructs cooperation.

Such intergroup threats may be based on real threats but may also be constructed by group leaders or members, and perceptions of threat are more valid than actual threats in how individuals respond to such perceptions (Stephan et al. 2015). For instance, if an individual believes that an outgroup and its members can threaten their job security, this belief will influence how that individual feels about the other group, even if their job situation is unaffected by members of the outgroup (Esses et al. 1998, 2001; Stephan et al. 2015). In this way, the same stimulus may be interpreted as a threat by some individuals and not by others. Rather, what is perceived as a threat is contingent upon an individual's perception and evaluation of a situation or target, which is biased by their social identification with certain groups.

Intergroup threat theory distinguishes between realistic threats and symbolic threats. Realistic threats are those that are perceived to threaten an ingroup's power, resources, or physical well‐being. Symbolic threats, on the other hand, are those that are perceived to threaten the integrity of an ingroup's meaning system, such as its values, identity, beliefs, or cultural norms (Rios et al. 2018). While these types of threats differ, they are often highly correlated (Kauff et al. 2013; Verkuyten 2009). An example is that of anti‐immigrant attitudes. Immigration is often perceived to constitute a realistic threat (jobs, security), but also symbolic threats, such as when different religions become immersed within a society. This was also how immigration was framed in the wake of Brexit (Abrams and Travaglino 2018).

In the present research, we do not separate between realistic and symbolic threats, theoretically or empirically. However, the perception of a threat is expected to be contingent upon identity with the ingroup (Rios et al. 2018). Thus, we suggest that information that the general population is positive to vaccines may be perceived by vaccine‐hesitant individuals as threatening to their position and identity. That is, the general population's opposite position to one's own could constitute a realistic threat, such as one's perceived physical well‐being from abstaining from vaccinations. Because realistic threats mainly concern power and material resources, manipulations of realistic threats often involve depicting an outgroup as competitive (Rios et al. 2018). Simply being informed about others' positions does not imply competition, and hence it is more likely that such information constitutes a symbolic threat that is threatening the ingroup's integrity, values, identity, and beliefs. Research shows that deviance between the values of the ingroup and the potentially threatening outgroup seems to induce symbolic threats (Rios 2013; Simpson et al. 2017).

Individuals who do not identify with this group will not perceive this information as threatening. Thus, only some people, namely those who identify as vaccine‐hesitant, will perceive population pro‐vaccination information as threatening. When experiencing a threat to their identity, individuals are likely to distance themselves from the other side, resulting in stronger anti‐vaccination attitudes (Renström et al. 2023).

### 2.2 The Mediating Role of Emotions

Emotions are important regulators of behavior that also influence cognitive processing (Frijda 1986). Several scholars have stressed the importance of emotions in political psychological research (Brader and Marcus 2013; Houghton 2009; Lambert et al. 2019; Renström and Bäck 2021; Renström et al. 2023). Emotions are important to human life in general and thus also to social and political life. Emotions inform individuals about a situation and prepare the body for a certain course of action (Frijda 1986). Emotions also affect cognitive processing, such as attention, information seeking, and reliance on heuristics and stereotypes (Brader and Marcus 2013). As such, emotions have an important place in explaining political behavior and attitudes. For instance, it is well‐established that emotions are important predictors in collective action (Goodwin et al. 2001; Gould 2009; Klandermans and van Stekelenburg 2013), as well as influencing how political information is processed and how political decisions are made (Brader 2006) and political attitudes in a broader sense (Brader and Marcus 2013; Lambert et al. 2019).

When it comes to epidemics and pandemics, emotions are highly present. Covid‐19 was quickly politicized, and threats about the disease or the economy were frames used by politicians (Green et al. 2020). Fear seems to be an important factor in vaccine hesitancy (Hornsey et al. 2018). Fear‐evoking disinformation about Covid‐19 and the vaccines against it was mainly distributed on social media, such as niche blogs and forums, but also on more readily available social media such as Twitter. Another important emotion is anger. Anger is evoked when people feel unfairly treated, and one important driving factor in vaccine hesitancy is that “the people” are tricked by Big Pharma's financial interests and therefore expose “the people” to risks on purpose, such as Covid‐19 (Hornsey 2021).

Generally, emotions can be divided into positive and negative emotions (Watson et al. 1988), and it is plausible to expect that when individuals experience intergroup threat, their emotional reactions will be negative. Drawing on research stressing the mediating role of emotions, we expect that when vaccine‐hesitant individuals experience a threat to their identity (when informed that population attitudes are positive to vaccination), they should react with negative emotions, which should increase anti‐vaccination attitudes. We therefore argue that the effect of threatening information about attitudes in the population will be mediated by negative emotions.

### 2.3 A Hypothesis About Intergroup Threat and Anti‐Vaccination Attitudes

**FIGURE 1:**
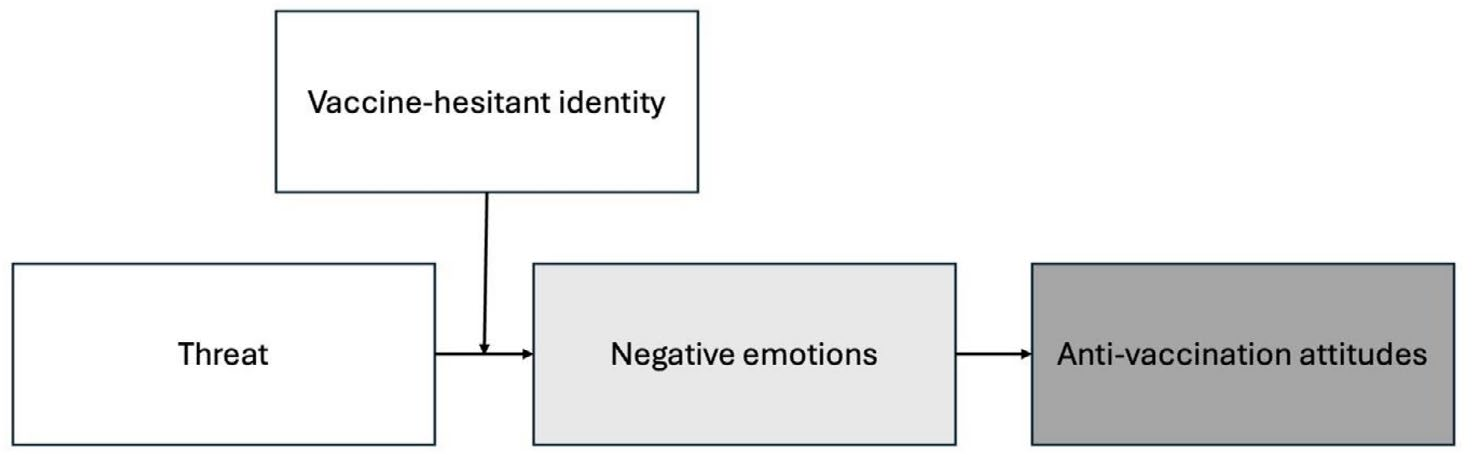
Theoretical model.

Drawing on the literature presented above, we make a theoretical contribution by focusing on the role of intergroup threat in anti‐vaccination attitudes. The theoretical model we propose is illustrated in Figure 1. We suggest that when individuals who identify as vaccine‐hesitant encounter data showing widespread public support for vaccination, this information may challenge their core beliefs and sense of self. In contrast, those who do not hold vaccine‐hesitant views are unlikely to find such information psychologically challenging. This creates a selective response pattern where pro‐vaccination population data specifically triggers defensive reactions among individuals who identify as vaccine‐hesitant. Faced with information that conflicts with their identity, these individuals will experience negative emotions and create greater psychological distance from pro‐vaccine perspectives, ultimately leading to more radical anti‐vaccination attitudes. We thus hypothesize that

*Individuals who identify as vaccine‐hesitant and who experience that their position is threatened will feel negative emotions, which will lead to higher anti‐vaccination attitudes*.

## 3 Methods and Data

### 3.1 Overarching Design and the Case of Sweden

We evaluate our hypothesis in the context of Sweden. Sweden constitutes an interesting case when it comes to exploring anti‐vaccination attitudes. Sweden is generally characterized by a high confidence in vaccines, and vaccination rates are generally high. For example, in the Swedish National Immunization Program, 97% of 2‐year‐olds followed the suggested vaccinations (Delilovic et al. 2022). More broadly, most developed countries report similarly high childhood vaccination coverage, indicating that vaccination is an accepted public health measure among the majority of the public (WHO 2025a, 2025b). In Sweden, most parents have a high confidence in the child immunization program, and when skepticism is expressed, it mainly concerns the HPV (Human papillomavirus), which is a relatively new addition to the program (Byström et al. 2020). However, when Covid‐19 hit, 40% of the Swedish population expressed doubts about vaccination during the first year of the pandemic (Lindvall and Rönnerstrand 2022). Hence, Swedes are generally more hesitant toward newer vaccines. To some extent, this hesitancy may be grounded in fear of consequences similar to those of the H1N1 vaccine, which led to an increase in narcolepsy among children and young adults (Läkemedelsverket 2020).

The two experiments presented in this article focus on exploring how individuals identifying as vaccine‐hesitant interpret information about vaccine attitudes in the population. Participants were informed that vaccine attitudes in the population were positive using fictive social media posts from a fictive opinion institute stating results from a new, large study. Based on intergroup threat theory (Stephan et al. 2015), we expected that vaccine‐hesitant individuals who were informed that vaccine attitudes are positive in the population would perceive this as a threat and react with negative emotions. The design was a between‐groups experiment where participants were randomly assigned to the experimental or control condition. The manipulation was presented as fictive Facebook posts.

We had three variations of stimulus intended to evoke perceptions of intergroup threat and six control variations. Participants were informed that they would be shown a social media post. The sender was a fictive opinion institute reporting on results from a recent, large study in Sweden. All posts started with the sentence “A new, large research study in Sweden showed…” and then followed information that the study was showing (1) that more people in Sweden have become positive toward vaccines; (2) more people choose to get vaccinated; or (3) more children follow the national immunization program. In the control condition, participants were informed by the same fictive opinion institute about a study showing that (1) more/fewer employees changed workplaces last year; (2) more/fewer Swedes started higher education last year; or (3) more/fewer Swedes exercise regularly. The three posts containing vaccine‐positive information were collapsed into one experimental condition, and the control posts were collapsed into a control condition.

## 4 Study 1

### 4.1 Participants and Procedure

In the first experiment, focusing on exploring the effect of intergroup threat on anti‐vaccination attitudes, 376 individuals participated. There were 202 (54%) women, 173 (46%) men, and 1 (0.3%) missing. Gender was recoded such that men = 1 and women = 0. Age ranged from 18 to 89 (*M* = 42, SD = 16).

The study was set up using the web survey tool *Qualtrics*, and data was collected via the survey company *Lysio*, which is a Swedish company that uses Norstat's online panels for data collection. Participants receive reimbursement of about 1€/15 min. The study was ethically approved by the Swedish Ethical Review Authority (2024‐06844‐02). Participants were first informed about ethics, the purpose and procedure of study participation, and asked to provide informed consent. They were then asked background and demographic questions, and then informed that they would be shown a post from Facebook. The post contained information about a recent survey, ostensibly posted by an opinion institute. The content claimed that the general population was positive toward vaccines and vaccinations.

After having seen the fictive post, participants were asked to rate to what extent they experienced different feelings while reading the text. Next, they were asked questions about their attitudes to vaccines and their own vaccine intentions. After this, they were debriefed and thanked for their participation.

### 4.2 Measures

The main dependent variable was *anti‐vaccination attitudes*, which were measured with 12 items from the Vaccination Attitudes Examination Scale (VAX; Martin and Petrie 2017). This scale is a comprehensive measure to assess anti‐vaccination attitudes across demographic groups and contexts and has been shown to have good reliability and validity. The scale consists of four subdimensions tapping into mistrust of vaccine benefits, worries over unforeseen future effects, concerns about commercial profiteering, and preference for natural immunity. Sample items are *I feel safe after being vaccinated* (reverse coded) and *Natural immunity lasts longer than a vaccination*. Responses were made on scales from 1 = Do not agree at all to 7 = Completely agree. We collapsed all items into an overall mean index (α = 0.89).

The key independent variable, *vaccine‐hesitant identity*, was measured with one question reading: “Sometimes we talk about different groups in society. To what extent would you say that you feel close to the group of vaccine‐hesitant individuals?” Answers ranged from 1 = Not close at all to 7 = Very close (see Appendix Table A1 for the distribution of the variable). This variable is included as a moderator in the analyses since we expect that individuals who identify as vaccine‐hesitant will react to the treatment with higher anti‐vaccination attitudes.

Our mediating variable was negative emotions. The question read: *When you read the post, to what extent did you experience these emotions?* Then a list of emotions followed, and answers were made on scales from 1 = Did not experience at all to 7 = Strong experience. The negative emotions were anger, frustration, indignation, shame, disgust, aversion, sadness, worriedness, anxiety, and unpleasantness (α = 0.96). All emotion items were presented in randomized order.

We also included a range of demographic and control variables. Research shows that women tend to be more hesitant than men, and younger people are more hesitant than older, as well as those with a lower education level (Latkin et al. 2022; Zintel et al. 2023). *Age* was measured in years using free text. *Gender* was measured with the response options man, woman, nonbinary, and do not want to answer. We recoded this into binary gender, where men = 1 and women = 0.^1^ *Education* was measured on a 5‐point scale from not completed basic schooling to doctoral degree.

We also controlled for ideology since it has been shown to be an important factor as the issue of Covid‐19 became highly politically polarized. Both Covid‐19 and anti‐vaccination sentiments in general have become contentious issues that are now politicized. Vaccine uptake in the US is highly correlated with political ideology, such that Democrats are more likely to get vaccinated than Republicans. In a study of 21 European countries, far‐right voters were less likely to get vaccinated than other voters (Backhaus et al. 2023). Also in Sweden, supporters of the radical right party, the Sweden Democrats, were among the most vaccine‐hesitant when Covid‐19 vaccines were introduced (Lindvall and Rönnerstrand 2022). *Ideology* was measured on a scale from 0 = Clearly to the left to 10 = Clearly to the right.

We also controlled for conspiracy beliefs since several studies showed that such beliefs about the pandemic and/or vaccines were a powerful predictor of vaccine hesitancy during Covid‐19 (Allington et al. 2021; McCarthy et al. 2021; Sallam et al. 2021) and lower compliance with safety measures (Chan et al. 2021). Anti‐vaccination attitudes also often co‐occur with other forms of conspiracy ideas (Batzdorfer et al. 2022; Gioia et al. 2023; Hornsey 2021; Jolley and Douglas 2014; Lewandowsky et al. 2013). Latkin et al. (2022) found that conspiracy beliefs were related to a lower likelihood of intending to get vaccinated against Covid‐19. *Conspiracy beliefs* were measured with five items from the Swedish SOM‐institute's survey (Strömbäck 2022). The question read: “The following statements concern your view of today's society. To what extent do you agree with these statements?” Two sample items are: *The official version that the authorities give about different events often hide the truth* and *Events that appear to lack a connection are often caused by secret activities*. Responses were made on 7‐point scales from 1 = Do not agree at all to 7 = Completely agree. The five items were collapsed into a mean index (α = 0.86).

Some studies show that individual differences may matter for a person's stance on vaccination, and we thus control for one such feature. The need for *cognitive closure* is a cognitive processing style striving for firm and clear answers and a dislike for ambiguity (Kruglanski and Webster 1996), which has been shown to influence vaccination attitudes. Individuals high in need for closure are motivated to reduce uncertainty by reaching definite answers quickly, and they do so with limited information search (Choi et al. 2008; Kruglanski 2004; Raglan et al. 2014). Hence, individuals high in need for closure do not process information about vaccines sufficiently thoroughly, and research shows that high need for closure is related to resistance against getting vaccinated (Cole et al. 2015; Solak et al. 2022). *Need for closure* was measured with four items. The question read: “These statements concern how you are as a person. Indicate to what extent you agree with these.” Two sample items are: *I don't like uncertain situations*, and *when I have made a decision, I feel relieved*. Responses were made on 7‐point scales from 1 = Do not agree at all to 7 = Completely agree. The four items were collapsed into a mean index (α = 0.65).

Finally, we included a manipulation check asking participants *to what extent do you perceive the content of the post that you read earlier as threatening?* Answers ranged from 1 = Not at all threatening to 7 = Very threatening.

## 5 Empirical Analyses

We first present descriptive analyses. The question assessing identity as vaccine‐hesitant was measured on a 7‐point scale, where lower values indicate dis‐identification and higher values indicate stronger identification. The mean of this scale was 2.89 (SD = 1.95). The mean of anti‐vaccination attitudes (i.e., VAX‐scale ranging from 1 to 7) was 3.70 (SD = 1.22). Hence, while vaccine‐hesitant identity was fairly low, anti‐vaccination attitudes were not extremely low, as the scale midpoint is 4. Table 1 presents a correlation matrix of all variables included in the study.

**TABLE 1:** Bivariate correlations between all variables, Study 1.

	Age	Gender	Education	Ideology	Conspiracy	NFC	ID	Emotions
Age								
Gender	0.07							
Education	−0.02	−0.11*						
Ideology	−0.04	0.18***	0.01					
Conspiracy	−0.20***	0.05	−0.08	0.17**				
NFC	−0.08	0.01	−0.09	0.05	0.41***			
Vaccine‐hesitant ID	−0.30***	0.09	−0.13*	0.15**	0.34***	0.16**		
Negative emotions	−0.31***	0.24***	−0.12*	0.20***	0.26***	0.13*	0.43***	
Anti‐vaccination attitudes	−0.29***	0.12*	−0.20***	0.15**	0.55***	0.18***	0.59***	0.37***

*Note:* Gender is coded 1 for men, 0 for women. *p < 0.5. **p < 0.01. ***p < 0.001.

As can be seen in Table 1, there were many of the expected correlations found in previous research, such that younger, women, less educated, and more right‐wing‐oriented individuals reported stronger anti‐vaccination attitudes compared to older, men, more educated, and left‐wing‐oriented individuals. Further, both conspiracy beliefs and need for closure were positively correlated with anti‐vaccination attitudes. The focal predictor variable vaccine‐hesitant identity, was also, as expected, strongly related to anti‐vaccination attitudes, such that a stronger vaccine‐hesitant identity was related to stronger anti‐vaccination attitudes.

To test if participants did indeed perceive the content of the post to be threatening, we ran a hierarchical regression model using the manipulation check item as the outcome variable and all the predictors in Model 1 and the interaction between threat and vaccine‐hesitant identity in Model 2. The results are shown in Table A2 in the Appendix. Importantly, the interaction was significant, indicating that participants who were exposed to the threat condition rated the content as more threatening when they scored higher on vaccine‐hesitant identity; see Figure A1 in the Appendix.

To better understand how identity as vaccine‐hesitant and intergroup threat shapes anti‐vaccination attitudes, we now turn to the experimental results. The experiment allows us to evaluate our hypothesis, which states that *vaccine‐hesitant individuals who experience that their position is threatened will feel negative emotions, which will lead to higher anti‐vaccination attitudes*. Before testing the full moderated mediation model, we show the effect of the treatment on emotions and how treatment and vaccine‐hesitant identity are related to emotional experience. We then test the full model using a moderated mediation analysis.

**FIGURE 2:**
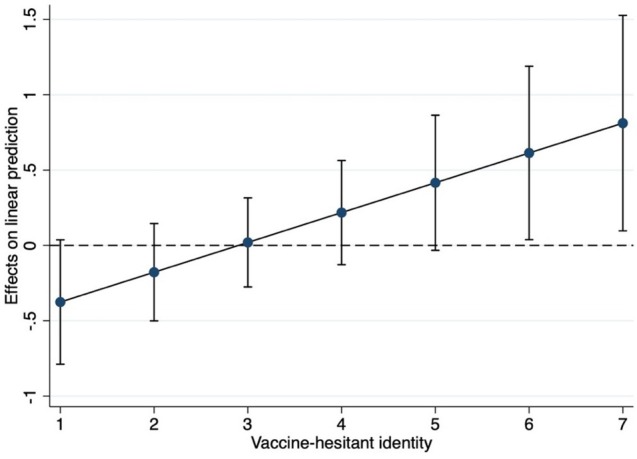
Marginal effects of vaccine‐hesitant identity in the threat condition on negative emotions, Study 1.

In Table 2, we present results from a hierarchical regression, testing the interaction between the threat condition and vaccine‐hesitant identity on the experience of negative emotions.^2^ We expected that the threat information would induce negative emotions for individuals identifying as vaccine‐hesitant. As can be seen in Model 2, there was a significant interaction between threat condition and vaccine‐hesitant identity. This is plotted in Figure 2.

**TABLE 2:** Regression models predicting negative emotions, Study 1.

	Model 1		Model 2	
	*B* (SE)	*p*	*B* (SE)	*p*
Age	−0.02 (0.00)	< 0.001	−0.02 (0.00)	< 0.001
Gender	0.70 (0.15)	< 0.001	0.68 (0.15)	< 0.001
Education	−0.13 (0.10)	0.20	−0.09 (0.10)	0.11
Ideology	0.05 (0.03)	0.10	0.04 (0.03)	0.12
Conspiracy	0.09 (0.06)	0.14	0.09 (0.06)	0.12
NFC	0.04 (0.07)	0.54	0.01 (0.07)	0.90
Vaccine‐hesitant ID	0.25 (0.04)	< 0.001	0.16 (0.27)	0.03
Threat condition^a^	−0.01 (0.15)	0.94	−0.57 (0.27)	0.04
Vaccine‐hesitant ID × Threat condition			0.20 (0.08)	0.01
Adj. *R* ^2^	0.28		0.29	

^a^ Threat condition is compared to the control condition.

As can be seen in Figure 2, there was a positive effect of the threat condition for individuals strongly identified as vaccine‐hesitant (rating 6 or 7 on the 7‐point scale) on negative emotions. Hence, receiving information that the population is positive to vaccines evokes negative emotions among individuals highly identified as vaccine‐hesitant. This could be taken as an indication that these people in fact perceive this information as threatening.

We now move on to test the full moderated mediation model (see Figure 1) using Hayes' (2018) Process macro.^3^ Table 3 shows the regression results from the moderated mediation predicting anti‐vaccination attitudes.

**TABLE 3:** Regression results from the moderated mediation model predicting anti‐vaccination attitudes, Study 1.

	*B* (SE)	*p*
Age	−0.01 (0.00)	0.001
Gender	0.14 (0.10)	0.17
Education	−0.21 (0.07)	0.002
Ideology	0.02 (0.02)	0.42
Conspiracy	0.42 (0.04)	< 0.001
NFC	−0.06 (0.05)	0.18
Threat condition	−0.11 (0.10)	0.27
Negative emotions	0.12 (0.03)	0.001
Adj. *R* ^2^	0.43	

**FIGURE 3:**
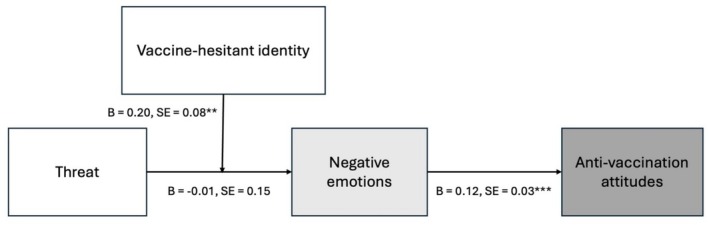
Results from the moderated mediation model, Study 1 (Threat condition predicting negative emotions, *β* = 0.00; vaccine‐hesitant ID × threat condition predicting negative emotions, *β* = 0.23; negative emotions predicting anti‐vaccination attitudes, *β* = 0.17).

As can be seen in Table 3, negative emotions predicted anti‐vaccination attitudes, such that a stronger experience of negative emotions was related to stronger anti‐vaccination attitudes.^4^ There was no direct effect of the threat condition on anti‐vaccination attitudes, *B* = −0,11, SE = 0,10, *p* = 0.27. However, the index of moderated mediation was significant, *B* = 0.02, BootSE = 0.01, LLCI = 0.003; ULCI = 0.05, indicating that the expected mediation of negative emotions was moderated by vaccine‐hesitant identity. Hence, individuals with stronger vaccine‐hesitant identity that are exposed to information suggesting that their attitude position is threatened experience negative emotions, which translates into stronger anti‐vaccination attitudes. The results from the moderated mediation model are presented in Figure 3.

## 6 Study 2

### 6.1 Participants and Procedure

Study 2 was performed to replicate the results from Study 1,^5^ using a larger sample to ensure sufficient power and increase generalizability. In the experiment, 698 Swedish participants finished the study. There were 356 (51%) women and 342 (49%) men. Gender was recoded such that men = 1 and women = 0. Age ranged from 18 to 89 (*M* = 49, SD = 16).

The study was set up using the web survey tool *Qualtrics*, and data was collected via the survey company *Lysio* again. Participants receive reimbursement of about 1€/15 min. The study was ethically approved by the Swedish Ethical Review Authority (2024‐06844‐02). The procedure was the same as in Study 1.

### 6.2 Measures

The main dependent variable was *anti‐vaccination attitudes*, again measured with the Vaccination Attitudes Examination Scale (VAX; Martin and Petrie 2017) (α = 0.92). The main independent variable, *vaccine‐hesitant identity*, was measured as in Study 1, with one question reading: “Sometimes we talk about different groups in society. To what extent would you say that you feel close to the group of vaccine‐hesitant individuals?” Answers ranged from 1 = Not close at all to 7 = Very close (see Appendix Table A1 for the distribution of the variable). The main experimental variable was potential *intergroup threat*. This was manipulated the same way as in Study 1. The mediating variable was negative emotions, assessed as in Study 1 (α = 0.97).

We also included the same demographic and control variables as we did in study 1, that is, age, gender, education, ideology, conspiracy beliefs (α = 0.88), and need for closure (α = 0.69).

Finally, we included a manipulation check asking participants *earlier in the study you were shown a post from Instagram. In your view, was the content of the post positive or negative?* Answers ranged from 1 = Very negative to 7 = Very positive.

### 6.3 Empirical Analyses

The mean value for identifying as vaccine‐hesitant was 1.91 (SD = 1.76), where 1 indicates the lowest level of identification and 7 the highest. This mean value is somewhat lower than the corresponding mean in Study 1 (2.89). For the VAX scale, which measures anti‐vaccination attitudes on a 7‐point scale (higher scores indicate stronger anti‐vaccination attitudes), the mean was 3.00 (SD = 0.73), also somewhat lower than in Study 1 (3.70).

Bivariate correlations between all study variables are presented in Table 4. Consistent with findings from Study 1, anti‐vaccination attitudes were more pronounced among younger individuals, those with lower levels of education, and those with more right‐wing political orientations, compared to older individuals, those more educated, and those with left‐wing orientations. Anti‐vaccination attitudes were also positively correlated with conspiracy beliefs, need for closure, and the key predictor, vaccine‐hesitant identity. A difference from Study 1 is that gender is not correlated with anti‐vaccination attitudes.

**TABLE 4:** Bivariate correlations between all variables, Study 2.

	Age	Gender	Educ.	Ideology	Consp.	NFC	ID	Emotions
Age								
Gender	0.06							
Education	−0.04	−0.01						
Ideology	0.09*	0.14***	−0.05					
Conspiracy	−0.04	0.01	−0.26***	0.21***				
NFC	−0.01	−0.12**	−0.14***	0.02	0.23***			
Vaccine‐hesitant ID	−0.14***	−0.02	−0.12**	0.21***	0.40***	0.10**		
Negative emotions	−0.18***	0.02	−0.15***	0.03	0.23***	0.16***	0.34***	
Anti‐vaccination attitudes	−0.10**	0.01	−0.23***	0.25***	0.55***	0.15***	0.69***	0.35***

*Note:* Gender is coded 1 for men, 0 for women. *p < 0.5; **p < 0.01; ***p < 0.001.

First, to test if participants did perceive the content of the post to be threatening, we ran a hierarchical regression model using the manipulation check item as the outcome variable and all the predictors in Model 1 and the interaction between threat and vaccine‐hesitant identity in Model 2. The results are shown in Table A3 in the Appendix. As can be seen, the interaction was significant, indicating that participants who were exposed to the threat condition rated the content as more negative if they scored higher on vaccine‐hesitant identity; see Figure A2 in the Appendix.

Our hypothesis states that *individuals who identify as vaccine‐hesitant who experience that their position is threatened will feel negative emotions, which will lead to higher* *anti‐vaccination attitudes*, which was supported in Study 1. We began by testing the effect of the treatment on emotions, as well as the interaction between treatment and vaccine‐hesitant identity. A hierarchical regression model was conducted, with results presented in Table 5.^6^

**TABLE 5:** Regression models predicting negative emotions, Study 2.

	Model 1		Model 2	
	*B* (SE)	*p*	*B* (SE)	*p*
Age	−0.01 (0.00)	< 0.001	−0.01 (0.00)	< 0.001
Gender	0.12 (0.09)	0.17	0.11 (0.09)	0.19
Education	−0.19 (0.07)	0.01	−0.20 (0.07)	0.01
Ideology	−0.02 (0.02)	0.15	−0.02 (0.02)	0.20
Conspiracy	0.06 (0.03)	0.09	0.06 (0.03)	0.10
NFC	0.12 (0.04)	0.01	0.12 (0.04)	0.00
Vaccine‐hesitant ID	0.19 (0.03)	< 0.001	0.11 (0.04)	0.01
Threat condition^a^	−0.09 (0.08)	0.30	−0.37 (0.13)	0.01
Vaccine‐hesitant ID × Threat			0.15 (0.05)	0.01
Adj. *R* ^2^	0.15		0.16	

^a^ Threat condition is compared to the control condition.

**FIGURE 4:**
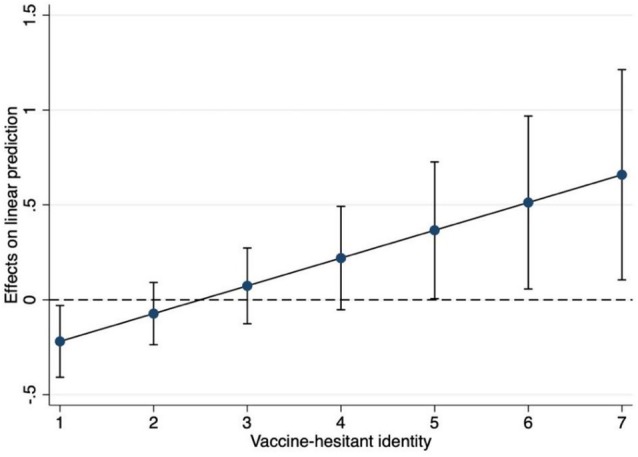
Marginal effects of vaccine‐hesitant identity in the threat condition on negative emotions, Study 2.

As shown in Model 2, the interaction between the threat condition and vaccine‐hesitant identity was significant, indicating that individuals who received the threat condition and scored higher on vaccine‐hesitant identity reported stronger negative emotions. These results are illustrated in Figure 4. Similar to the results found in Study 1, there was a positive effect of the threat condition for individuals strongly identified as vaccine‐hesitant on negative emotions.

To test the full hypothesis and whether the results from Study 1 would replicate, we conducted a moderated mediation analysis using Hayes' (2018) macro. The regression results are presented in Table 6. Negative emotions^7^ predicted stronger anti‐vaccination attitudes; however, the threat condition itself had no direct effect on anti‐vaccination attitudes, *B* = −0.04, SE = 0.08, *p* = 0.60.

**TABLE 6:** Regression results from the moderated mediation model predicting anti‐vaccination attitudes, Study 2.

	*B* (SE)	*p*
Age	−0.01 (0.00)	0.02
Gender	−0.03 (0.08)	0.67
Education	−0.15 (0.07)	0.03
Ideology	0.08 (0.02)	< 0.001
Conspiracy	0.40 (0.03)	< 0.001
NFC	0.01 (0.04)	0.81
Threat condition	−0.05 (0.08)	0.54
Negative emotions	0.23 (0.04)	< 0.001
Adj. *R* ^2^	0.39	

**FIGURE 5:**
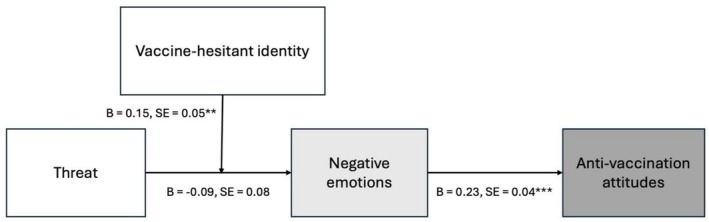
Results from the moderated mediation model, Study 2 (Threat condition predicting negative emotions, *β* = −0.04; vaccine‐hesitant ID × threat condition predicting negative emotions, *β* = 0.19; negative emotions predicting anti‐vaccination attitudes, *β* = 0.21).

**FIGURE A1:**
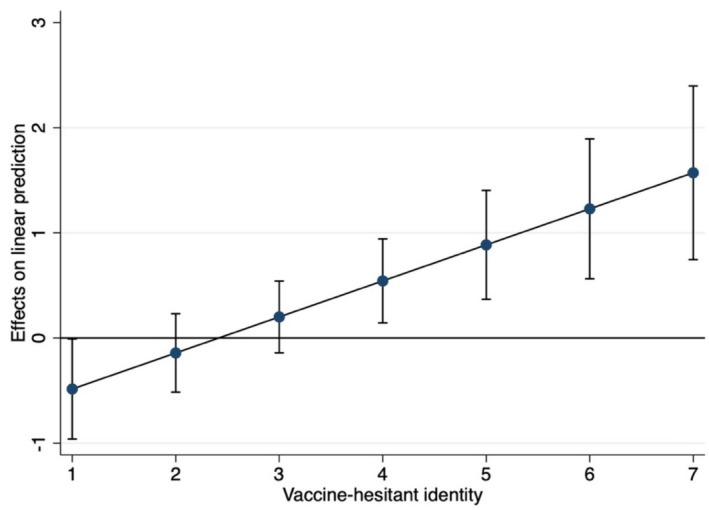
Marginal effects of vaccine‐hesitant identity in the threat condition on the manipulation check, Study 1.

**FIGURE A2:**
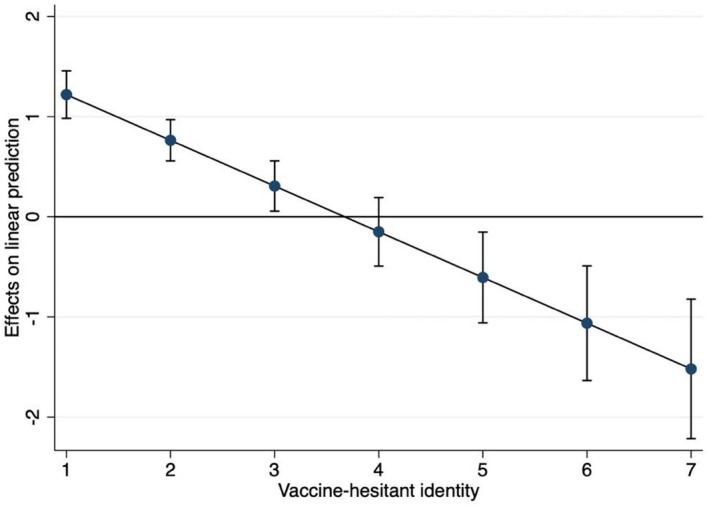
Marginal effects of vaccine‐hesitant identity in the threat condition on the manipulation check, Study 2.

Importantly, the index of moderated mediation was significant, *B* = 0.03, BootSE = 0.02, LLCI = 0.001; ULCI = 0.08, suggesting that the mediating effect of negative emotions on anti‐vaccination attitudes is moderated by vaccine‐hesitant identity. In other words, individuals with a stronger vaccine‐hesitant identity who were exposed to information that threatened their attitudinal position experienced stronger negative emotions, which in turn led to stronger anti‐vaccination attitudes. This replicates the findings from Study 1. See Figure 5 for the results from the moderated mediation model.

## 7 Discussion

Vaccine hesitancy and decreasing rates of vaccination are among the most pressing global challenges of our time (WHO 2019). Anti‐vaccination attitudes and the associated refraining from getting vaccinated have led to new outbreaks of diseases and even deaths that would be vaccine‐preventable (Phadke et al. 2016). For instance, several outbreaks of measles have been documented in the U.S., and the number of cases seems to increase over the years. Importantly, most of the reported cases are unvaccinated individuals (CDC 2025). Much of the previous research has focused on socioeconomic and sociodemographic factors and health literacy (Giambi et al. 2018; Guay et al. 2019; Montagni et al. 2021). Psychological factors also play a significant role, as does distrust in public authorities and low confidence in either the vaccine itself or the healthcare providers administering it. Additionally, hesitancy may stem from complacency, where individuals do not perceive a need for vaccination or fail to recognize its value, as well as issues related to convenience, such as limited access to vaccination services (Larson et al. 2014). Hence, it is of the highest relevance to understand the psychological underpinnings of anti‐vaccination attitudes. Thus, the aim of the present research was to contribute to the understanding of how social identity is related to anti‐vaccination attitudes by exploring if information about others' position on this issue could be perceived as an intergroup threat by individuals strongly identifying as vaccine‐hesitant.

Identifying with social groups is important to individuals, and such identities become defining parts of the view of oneself that also imply comradery and support within the group (Tajfel and Turner 2004). Previous research shows that social identity constitutes an important bonding principle between individuals sharing anti‐vaccination attitudes (Motta et al. 2023). When this identity is perceived to be under threat, for instance, from information that most other people in the population do not share the own position, this may lead to experiences of negative emotions that could translate into anti‐vaccination attitudes. By adopting more radical anti‐vaccination attitudes, individuals who perceive an intergroup threat may increase the psychological distance to the threatening group, which functions to solidify group boundaries and strengthen one's own social identity, in line with intergroup threat theory (Iyengar et al. 2019; Kruglanski et al. 2019; Stephan et al. 2015; Renström et al. 2023). Thus, we hypothesized that individuals who identify as vaccine‐hesitant would perceive information that the general population was vaccine‐positive as threatening, leading to negative emotions and subsequently, stronger anti‐vaccination attitudes. We evaluated this hypothesis using two experiments in Sweden.

In our experiments, we presented participants with fictive information describing that the general population was positive to vaccines and vaccination, intended to constitute an intergroup threat for those feeling strongly identified as vaccine‐hesitant. In a control condition, participants were shown unrelated information. In both experiments, we find that individuals who identify as vaccine‐hesitant seem to interpret this information as threatening, as it evoked negative emotions among these people. We further find that these negative emotions seem to translate into anti‐vaccination attitudes. That is, the stronger the emotional reaction to the threatening information is, the stronger an individual's anti‐vaccination attitudes are.

We did not find a main effect of treatment on anti‐vaccination attitudes, which indicates that interpretation of this type of information is contingent upon individual differences, in this case identification as vaccine‐hesitant. However, this also indicates that the manipulation was relatively subtle. For instance, we did not explicitly state that the population's positivity to vaccines and vaccination was in fact threatening to those with a strong vaccine‐hesitant identity. Nonetheless, we would not expect a main effect of treatment, as it can be expected that information about the population's position on vaccines and vaccination should be diametrically differently processed by those with a low vaccine‐hesitant identity (i.e., population positivity is own support) and those with a strong vaccine‐hesitant identity (i.e., population positivity is threatening), ultimately leading to null effects.

In the experiments, we did not empirically separate between realistic and symbolic threats as they are presented in intergroup threat theory (Stephan et al. 2015). Based on theory, we would argue that the type of threat manipulated in our studies is symbolic. It is unlikely that the participants believe that others' opposing attitudes would have consequences in terms of security or even bodily autonomy since the choice to get vaccinated is not regulated by authorities. Thus, it is more plausible that the participants experienced the information as threatening to their values, beliefs, and personal integrity.

Hence, communication about vaccine positions as positive in the public seems to be polarizing in the sense that it leads vaccine‐hesitant individuals to reinforce their previous attitudinal position. These results are important since they highlight the complexity in communicating information about vaccination rates and the necessity to carefully formulate such communication to avoid triggering identity concerns. Social group memberships are central to an individual's self‐definition, thereby influencing cognitions and behavior (Tajfel and Turner 2004). For those who are emotionally and psychologically attached to the group membership of “vaccine‐hesitants” as it satisfies fundamental human needs, a change of opinion may be more difficult than for those who are truly invested in the issue, as identity concerns may function to strengthen and crystallize attitudes (Howe and Krosnick 2017). Hence, instead of focusing on communicating widespread vaccination norms, messages could highlight how vaccines directly benefit loved ones (i.e., the ingroup). And personal stories from former hesitants (the ingroup), who overcame their hesitations and chose to vaccinate, may be more persuasive than statistical arguments in encouraging vaccine acceptance in this group.

Another venue for communicating about vaccines is to consider the complex social identities that people have. That is, individuals are not members of a single social group but multiple groups (Brewer 1991; Roccas and Brewer 2002). This suggests that activating other group memberships may be key to reducing anti‐vaccination attitudes. This means that such communication is highly delicate. If an individual feels that their position is threatened, their social identification with that group may strengthen, leading to the opposite effect. In line with self‐categorization theory, when a social identity is salient, the self becomes depersonalized in the way that the individual becomes one with the prototype of the group (Hogg 1992; Turner et al. 1987; Postmes and Spears 1998), which means that it becomes more difficult to see oneself as an individual with other characteristics. That is, if an individual is fully immersed in the group, change is unlikely (see e.g., Abrams and LaLot (2025) on intergroup communication).

In our theoretical framework we have focused on intergroup threat, intergroup emotions, and attitudes. While the results align with the suggested theoretical model, there may be alternative explanations to the results that warrant mentioning. For instance, motivated reasoning (Kunda 1990) may account for a reinforcement of an attitudinal position when exposed to contradictory information as a way to avoid cognitive dissonance. If people were normatively or informationally influenced by the information that the general population was positive to vaccines, this may have led to cognitive dissonance among those identified as vaccine‐hesitant.

Some limitations are worth noting. Our main independent variable was identification with vaccine‐hesitants, which was measured with a single item asking participants if they felt close to the group of “vaccine‐hesitants.” While this item does not measure social identification as it is traditionally measured, we believe that this variation, which is a bit “softer” is beneficial, especially in a country such as Sweden, where vaccine‐hesitancy is generally frowned upon. In a qualitative study performed in Sweden, the authors found that declining vaccination was associated with stigma and that the participants perceived the norm in Sweden to be to just accept recommendations from authorities. In relation to this stigma, many of the participants in this study claimed to have “safe communities” where they felt they could openly share their attitudes (Herzig van Weer and Ström 2024). This indicates that factors such as social identity and perceived social support from one's ingroup may be highly important in this context.

Indeed, the mean of the identity item was relatively low, indicating that this identity is relatively unusual in Sweden. We chose this measure instead of measuring strict identity, as we believe this might have resulted in even lower values and potential problems with skewness and floor effects. Moreover, while ingroup identification often is operationalized as the importance or centrality of one's ingroup to the self‐concept (Rios et al. 2018), it can also be operationalized in terms of attachment to a group, such as feeling strong ties to the group (Roccas et al. 2006). This latter operationalization comes closer to the measure we used in this article. Another potential problem with the phrasing of this item, where we ask how “close” the participant feels to the group “vaccine‐hesitants” is that they might have interpreted “close” rather in terms of attitude similarity than “attachment.” Thus, we encourage future research to use other measures in contexts where this particular social identity is more socially accepted.

An associated limitation is that of generalizability. Because Sweden has the above‐described characteristics, the results may not directly translate to other contexts. For instance, if the social identity is more accepted, there may be less need to defend it against outside threats. It could also be that other unmeasured variables influence the results, such as trust in authorities and perceived stigma of being vaccine‐hesitant. These effects may be lower in other national contexts. Thus, we encourage researchers to explore the suggested mechanisms in other contexts and from a comparative perspective.

We have here focused on anti‐vaccination attitudes, that is, attitudes toward vaccines and vaccination, rather than the actual behavior of those who are vaccine‐hesitant. However, in the Appendix, we also present analyses from Study 2 with vaccine intentions in a future pandemic/epidemic as the outcome variable. These analyses show a strong association between anti‐vaccination attitudes and vaccine intentions, consistent with previous research (Biella et al. 2023).

Another limitation is that the experimental context does not allow for conclusions about long‐term effects. As we measured anti‐vaccination attitudes in the same survey at the same time, we cannot say to what extent the observed attitude changes are stable. Most likely, the effects are temporary. Nonetheless, the aim of this type of experiment is rather to explore potential mechanisms. Given the amount of information that people are exposed to in their everyday lives, it is possible that the effects observed in this type of relatively artificial experimental setting have larger effects outside the experimental context. In relation to this, the effects of the moderated mediation were relatively small. As this type of stimuli material can be expected to be quite weak, that is not surprising. Also, there was relatively little variation in the moderator variable of vaccine‐hesitant identity, which further contributes to difficulties in finding strong statistical effects. It should also be noted that we here used online panels to collect data. Since such panels are often biased toward progressive, higher‐educated samples, it is possible that the effects are underestimated compared to a truly random population sample. With this in mind, the fact that we do find treatment effects across two studies, nonetheless provides relatively strong evidence of the suggested mechanisms. We encourage future research to explore the suggested mechanisms in more natural settings and with other types of data. This would also be beneficial for external validity. While experiments are good at establishing causality, the experimental context may be quite artificial. That is, it is unlikely that individuals are exposed to a single source of information in real life.

We have argued that the experience of negative emotions leads to variations in anti‐vaccination attitudes, but we cannot rule out reverse causation. That is, while it seems logical that emotions influence attitudes, it could be the other way around, that attitudes lead to emotional experiences. Another related caveat is common method variance, such that because emotions and attitudes were measured in close succession, responses might be artificially related because of the method, not because the constructs are truly related.

Finally, one important remaining question is the mechanism as to why vaccine‐hesitant individuals experience pro‐vaccination norms as threatening. One possible mechanism relates to the concept of “healthism”, which captures the belief that health is a personal responsibility achieved through the individual's lifestyle, that is, individuals who are vaccine‐hesitant may be more likely to adhere to healthism (Milionis et al. 2023; Kirbiš 2023), and so being “told” that they should get vaccinated by society feels intrusive and controlling. Future research should explore this potential mechanism.

To conclude, this research shows that social identity is an important predictor of anti‐vaccination attitudes, which could also influence cognitive processing. This is important, because if such attitudes are mainly rooted in an identity position, they are difficult to influence. Attempts to change or influence such attitudes may rather be perceived as an attack against one's identity, the core of who one is, and thus, resistance may instead increase.

## Author Contributions

E.A.R. and H.B. main idea. E.A.R. draft and preliminary analyses. All authors have been involved in reading and writing, and revising the manuscript.

## Funding

This work was supported by the Swedish Research Council under Grant number 2022‐06316.

## Conflicts of Interest

The authors declare no conflicts of interest.

## Data Availability

The data that support the findings of this study are available on request from the corresponding author. The data are not publicly available due to privacy or ethical restrictions.

## Endnotes

^1^
Only one person answered “other” and was removed from the analyses with gender.

^2^
As the predictor variables were highly correlated, we also ran a multicollinearity analysis, which showed that none of the VIF values exceeded 5, and the mean VIF value was 1.88.

^3^
We used Hayes' Process macro for SPSS, model 7.

^4^
We used a negative emotions index composed of varying emotions. To explore the different emotions further, we ran a principal component analysis, which indicated a single factor, and we correlated each emotion with the VAX‐scale (see Tables A4 and A5 in the Appendix). The correlation coefficients were of equivalent size and all positively correlated to anti‐vaccination attitudes.

^5^
In the Appendix Table A6, we also present analyses using vaccine intentions in a future pandemic/epidemic as the outcome variable.

^6^
Again, the multicollinearity analysis indicated no problems with VIF values lower than 4, and mean VIF = 1.70.

^7^
As in Study 1, a principal component analysis extracted one factor, and all correlation coefficients between each negative emotion and anti‐vaccination attitudes was of equivalent size, positive, and significant; see Tables A4 and A5 in the Appendix.

## Appendix

**TABLE A1:** Frequency and percent for the vaccine‐hesitant identity item, Studies 1 and 2.

Scale (1–7)	Study 1		Study 2	
	Frequency	Percent	Frequency	Percent
1	137	36.4	466	66.8
2	50	13.3	72	10.3
3	50	13.3	32	4.6
4	49	13.0	52	7.4
5	42	11.2	35	5.0
6	24	6.4	13	1.9
7	21	5.6	22	3.2
Valid total	373	99.2	692	99.1
Missing	3	0.8	6	0.9
Total	376	100.0	698	100.0

**TABLE A2:** Predicting the manipulation check, Study 1.

	Model 1		Model 2	
	*B* (SE)	*p*	*B* (SE)	*p*
Age	−0.02 (0.01)	0.002	−0.02 (0.01)	0.004
Gender	0.71 (0.18)	< 0.001	0.68 (0.17)	< 0.001
Education	−0.01 (0.12)	0.923	0.06 (0.12)	0.586
Ideology	0.05 (0.03)	0.122	0.05 (0.03)	0.160
Conspiracy	−0.01 (0.07)	0.901	−0.01 (0.07)	0.937
NFC	−0.00 (0.08)	0.959	−0.07 (0.08)	0.438
Vaccine‐hesitant ID	0.34 (0.05)	< 0.001	0.19 (0.06)	0.003
Threat condition^a^	0.15 (0.18)	0.407	−0.83 (0.31)	0.009
Vaccine‐hesitant ID × Threat condition			0.34 (0.09)	< 0.001
Adj. *R* ^2^	0.23		0.26	

*Note:* The outcome variable is to what extend do you perceive the content of the post you read earlier as threatening? Answers ranged from 1 = Not at all threatening to 7 = Very threatening. Gender is coded 1 for men, 0 for women.

**TABLE A3:** Predicting the manipulation check, Study 2.

	Model 1		Model 2	
	*B* (SE)	*p*	*B* (SE)	*p*
Age	0.04 (0.00)	0.285	0.00 (0.00)	0.217
Gender	−0.00 (0.11)	0.968	0.01 (0.11)	0.946
Education	0.13 (0.10)	0.168	0.13 (0.09)	0.144
Ideology	0.00 (0.02)	0.853	−0.00 (0.02)	0.913
Conspiracy	−0.04 (0.04)	0.348	−0.03 (0.04)	0.430
NFC	0.11 (0.05)	0.043	0.10 (0.05)	0.061
Vaccine‐hesitant ID	−0.24 (0.04)	< 0.001	−0.00 (0.05)	0.925
Threat condition^a^	0.81 (0.11)	< 0.001	1.67 (0.16)	< 0.001
Vaccine‐hesitant ID × Threat condition			−0.46 (0.08)	< 0.001
Adj. *R* ^2^	0.14		0.20	

*Note:* The outcome variable is earlier in the study you were shown a post from Instagram. In your view, was the content of the post positive or negative? Answers ranged from 1 = Very negative to 7 = Very positive. Gender is coded 1 for men, 0 for women.

**TABLE A4:** Principal component analysis on the negative emotions, Studies 1 and 2.

	Study 1	Study 2
	Component	Component
	1	1
Anger	0.87	0.86
Frustration	0.85	0.80
Indignation	0.85	0.81
Shame	0.81	0.81
Disgust	0.86	0.79
Aversion	0.88	0.88
Sadness	0.87	0.89
Worry	0.83	0.88
Upsetness	0.86	0.88
Anxiety	0.86	0.89
Unpleasantness	0.89	0.92

**TABLE A5:** Correlations between anti‐vaccination attitudes and the negative emotions, Studies 1 and 2.

	Study 1	Study 2
Anger	0.31***	0.28***
Frustration	0.33***	0.29***
Indignation	0.34***	0.29***
Shame	0.30***	0.28***
Disgust	0.32***	0.34***
Aversion	0.32***	0.34***
Sadness	0.34***	0.29***
Worry	0.31***	0.28***
Upsetness	0.32***	0.31***
Anxiety	0.31***	0.28***
Unpleasantness	0.33***	0.29***

*Note:* ****p* < 0.001.

### Analyses Using Vaccine Intention as the Outcome, Study 2

In Study 2, we also included a measure on vaccine intentions as an outcome variable. Participants were asked *In the event of a future epidemic or pandemic, how likely is it that you would get vaccinated if a vaccine were available free of charge and recommended by the authorities?* Answers were given on a scale from 1 = Very unlikely to 7 = Very likely. The mean in the whole sample was 5.64 (SD = 2.04). Bivariate correlations between vaccine intention and all other variables are presented in Table A6.

The regression results from the moderated mediation model predicting vaccine intention are presented in Table A7. Negative emotions predicted weaker vaccine intention; however, the threat condition itself had no direct effect on vaccine intention, *B* = −0.13, SE = 0.14, *p* = 0.36.

The index of moderated mediation was significant, *B* = −0.04, BootSE = 0.02, LLCI = −0.09; ULCI = −0.001, suggesting that the mediating effect of negative emotions on vaccine intention is moderated by vaccine‐hesitant identity. In other words, individuals with a stronger vaccine‐hesitant identity who were exposed to information that threatened their attitudinal position experienced stronger negative emotions, which in turn led to weaker vaccine intention in the event of a future epidemic or pandemic.

**TABLE A6:** Bivariate correlations between vaccine intention and all other variables, Study 2.

	Vaccine intention
Age	0.20***
Gender	0.03
Education	0.16***
Ideology	−0.18***
Conspiracy	−0.38***
NFC	0.02
Vaccine‐hesitant ID	−0.60***
Negative emotions	−0.24***
Anti‐vaccination attitudes	−0.64***

*Note:* Gender is coded 1 for men, 0 for women. ****p* < 0.001.

**TABLE A7:** Regression results from the moderated mediation model predicting vaccine intention, Study 2.

	*B* (SE)	*p*
Age	0.02 (0.00)	< 0.001
Gender	0.35 (0.15)	0.02
Education	0.23 (0.12)	0.07
Ideology	−0.12 (0.03)	< 0.001
Conspiracy	−0.46 (0.06)	< 0.001
NFC	0.24 (0.07)	< 0.001
Threat condition	−0.13 (0.14)	0.36
Negative emotions	−0.23	< 0.001
Adj. *R* ^2^	0.22	

*Note:* Gender is coded 1 for men, 0 for women.
